# Supplementary material for: Electroluminescence and current–voltage measurements of single-(In,Ga)N/GaN-nanowire light-emitting diodes in a nanowire ensemble
Source: Beilstein J Nanotechnol. 2019 Jun 5;10:1177–87. doi: 10.3762/bjnano.10.117 (PMC6604741; doi:10.3762/bjnano.10.117)
Supplement: File 1 — Additional experimental data. [file Beilstein_J_Nanotechnol-10-1177-s001.pdf]

BEILSTEIN JOURNAL OF NANOTECHNOLOGY

# Supporting Information

for

# Electroluminescence and current–voltage measurements of single-(In,Ga)N/GaN-nanowire light-emitting diodes in a nanowire ensemble

David van Treeck, Johannes Ledig, Gregor Scholz, Jonas Lähnemann, Mattia Musolino, Abbes Tahraoui, Oliver Brandt, Andreas Waag, Henning Riechert and Lutz Geelhaar

*Beilstein J. Nanotechnol.* **2019,** *10,* 1177–1187. doi:10.3762/bjnano.10.117

# Additional experimental data

License and Terms: This is a supporting information file under the terms of the Creative Commons Attribution License (http://creativecommons.org/licenses/by/4.0). Please note that the reuse, redistribution and reproduction in particular requires that the authors and source are credited.
The license is subject to the *Beilstein Journal of Nanotechnology* terms and conditions: (https://www.beilstein-journals.org/bjnano)

Due to the resolution limit of the scanning electron microscope (SEM) and the restricted angle between sample and probe tip, it was not possible to identify by SEM the exact number of contacted NWs below the tungsten tip for the various measurements presented in the paper. However, slightly moving the probe tip sideways across the tips of the emerging NWs, we discovered that emission bands vanish in the live-monitored electroluminescence (EL) spectrum while eventually new bands appear at different emission energies. This suggests that the emission bands in the spectra of the different measurement points shown in Figure 2 of the main paper originate from single NWs.

Indeed, analyzing *I*–*V* and EL behavior of measurement point D for different applied currents as shown in Figure S1a, we find that the *I*–*V* behavior shows a sudden increase in voltage and a decrease in slope between 80 and 85 nA, when one of the emission bands in the EL spectrum (inset) disappears due to a slight movement of the tip. Figure S1b shows that the single emission bands in the EL spectra for 80 and 85 nA can be well fitted by Lorentzian curves and that, indeed, the emission band for NW 3 has vanished for 85 nA.

The decrease in slope of the current–voltage curve corresponds to an increase in the total series resistance of the contacted NW cluster. This can be explained considering that a cluster of three parallel NWs (assuming each NW has a similar resistance) has a total resistance lower by a factor of 1.5 than two parallel connected NWs. And indeed, extracting the series resistance of the two current regions, below 80 nA (65–80 nA) and above 85 nA (85–95 nA) by analyzing the slope, one obtains a ratio of 1.7, hence, close to the calculated one.

In summary, this example shows that the single emission bands in the EL spectra of common measurement points as shown in Figure 2 in the main text is most likely EL of single NWs.

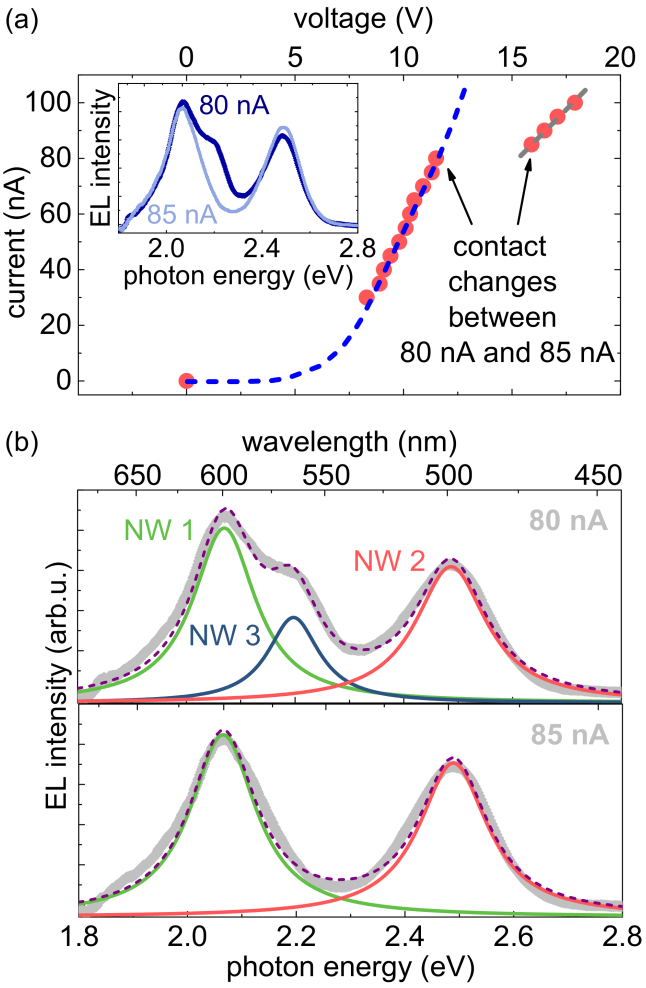


**Figure S1:** (a) *I*–*V* behavior of measurement point D (cf. Figure 2 in the main paper) as a function of the applied current (for clarity reasons the applied current is plotted vertically). The dashed lines serve as a guide to the eye. The inset shows the EL spectra for applied currents of 80 nA and 85 nA. (b) Fits of the single emission band profiles in EL spectra for applied currents of 80 nA and 85 nA by Lorentzian curves. The dashed lines show the respective cumulative fits resulting from the single fits of the line profiles.
